# Antiviral Activity of *Ganoderma lucidum* Polysaccharide Against Pseudorabies Virus via Reducing Virus Adsorption and Replication In Vitro

**DOI:** 10.1155/tbed/5920730

**Published:** 2026-11-14

**Authors:** Jianbin Wang, Xinrui Cai, Song Gao, Changchao Huan

**Affiliations:** ^1^ College of Veterinary Medicine Yangzhou University Yangzhou China yzu.edu.cn; ^2^ Jiangsu Co-Innovation Center for Prevention and Control of Important Animal Infectious Diseases and Zoonoses Yangzhou University Yangzhou China yzu.edu.cn; ^3^ Key Laboratory of Avian Bioproduct Development Ministry of Agriculture and Rural Affairs Yangzhou University Yangzhou China yzu.edu.cn; ^4^ Institutes of Agricultural Science and Technology Development Yangzhou University Yangzhou China yzu.edu.cn

**Keywords:** adsorption, *Ganoderma lucidum* polysaccharide, pseudorabies virus, replication

## Abstract

Since the emergence of highly virulent pseudorabies virus (PRV) strains in Chinese swine, outbreaks have resulted in considerable economic consequences for the livestock industry. The limited protective effect of current vaccination programs highlights the necessity of identifying alternative antiviral approaches. *Ganoderma lucidum* is one of the traditional Chinese medicines, and *Ganoderma lucidum* polysaccharide (GLP) is recognized as a key bioactive component. Although GLP exhibits multiple pharmacological properties, its antiviral activity specifically against PRV has not been previously documented. Our findings demonstrate that GLP suppresses PRV infection, as evidenced by western blot, indirect immunofluorescence assay (IFA), and 50% tissue culture infective dose (TCID_50_) analyses. Stage‐specific analyses revealed that GLP mainly affected the initial viral attachment process and subsequent intracellular amplification of PRV. Therefore, GLP could be a new therapeutic agent for preventing PRV infection.

## 1. Introduction

Aujeszky’s disease (AD), also known as pseudorabies, is caused by pseudorabies virus (PRV), which is part of the Herpesviridae family, Alphaherpesvirinae subfamily, and Varicellovirus genus [1]. It consists of four main components, the nucleus (double‐stranded DNA), capsid, tegument, and envelope [2]. PRV causes neurological disorders in newborn piglets and reproductive failure in sows [3]. Beyond swine, PRV has demonstrated a broad host range among several mammalian species, including cattle, sheep, cats, dogs, mink, and rodents. In these nonnatural hosts, PRV infection is marked by intense itching, a brief disease course, and swift mortality [4]. Historical records indicate that PRV was initially identified in domestic cats in eastern China during the late 1940s, followed by reports in pigs, cattle, goats, and other animals [5]. Due to inadequate detection technology and insufficient biosafety protocols in the pig industry, PR was widespread in many parts of China during the early 1980s [5]. From the 1990s to the end of 2011, over 80% of pigs in China received the Bartha‐K61 vaccine, originally brought from Hungary in the 1970s, effectively controlling pseudorabies. However, pseudorabies reemerged in late 2011 [6]. Moreover, PRV was isolated from human cases of acute encephalitis [7], and since 2017, China has reported 25 cases of PRV infections in humans, suggesting a major risk of PRV being transmitted from pigs to humans, thereby posing an increasing threat to human and public health [8].

In recent years, natural polysaccharides have emerged as promising antiviral candidates, such as antiviral activities, making them an excellent alternative. They are crucial for the metabolic processes in diverse biological systems, including plants, microorganisms, and animals, and can be directly extracted. Polysaccharides have promoted interest in polysaccharide‐based antiviral development because of their therapeutic benefits, low toxicity, and accessibility, making them promising candidates for future antiviral drugs [9]. Studies in pharmacology have demonstrated that the modified polysaccharide possesses a range of biological functions, such as antioxidant, antitumor, immune regulation, and antiviral, antibacterial, and anticoagulant effects in vitro. Over 300 types of natural polysaccharide compounds have been discovered so far. These compounds are found in plants, animals, and microorganisms, and they perform various physiological roles. During the 1950s, polysaccharides gained attention in pharmacological research, particularly in immunopharmacology, because of their immunoregulatory and antitumor properties [10].

*Ganoderma lucidum*, recognized as a white‐rot fungus, has been traditionally valued in China for its health‐promoting and life‐extending properties [11]. Around 400 unique bioactive substances are found in the mycelia, fungi, and *Ganoderma lucidum*, including polysaccharides, nucleotides, steroids, triterpenoids, fatty acids, sterols, proteins/peptides, and trace elements. Among them, *Ganoderma lucidum* polysaccharide (GLP) is the major bioactive component [12], which has antioxidant activity, liver protection, anticancer activity, immune regulation, and antibacterial activity [13]. In addition, GLP can act on several pathways, which can have a positive effect on cancer treatment. The application of GLP to cervical cancer cells caused the decline in phosphorylated‐JAK and phosphorylated‐STAT5 levels, highlighting its inhibitory role on the JAK/STAT5 pathways [14]. GLP hinders lung cancer progression by promoting MDSC differentiation and reducing their accumulation through the CARD9‐NF‐κB‐IDO pathway [15].

Although numerous bioactive compounds from *Ganoderma lucidum* (e.g., ganoderic acids, ganodermanondiol, and ganodermanontriol) exhibit broad‐spectrum antiviral activity against pathogens including enterovirus 71, dengue virus, HSV, HIV, H1N1, H5N1, and coronavirus [16, 17], no studies have investigated the potential of GLP to inhibit PRV. Therefore, this study aims to be the first to investigate this activity. Thus, our study assessed the antiviral effects of GLP on PRV in vitro and demonstrated that GLP primarily inhibits PRV attachment and replication. Our results can provide insights for using GLP, particularly in antiviral applications.

## 2. Materials and Methods

### 2.1. Cells and Virus

PK‐15 cells were cultured in Dulbecco’s Modified Eagle Medium (DMEM; Sigma) supplemented with 5% fetal bovine serum (FBS; Lonsa, Uruguay). All cells were propagated at 37°C in a humidified incubator with 5% CO_2_. The strain PRV XJ5, employed for the infections, was archived in the innovation team for prevention and control of swine and poultry important infectious diseases laboratory.

### 2.2. Chemicals, Reagents, and Antibodies

GLP (purity ≥98% as determined by UV) was from Yangling Ci Yuan Biotechnology Co., Ltd. (Shanxi, China). A stock solution of 50 mg/mL was prepared in phosphate‐buffered saline (PBS), aliquoted, and stored at −20°C. A citric acid solution (40 mM citric acid, 135 mM NaCl, and 10 mM KCl) was prepared, adjusted to pH 3.0, and stored at 4°C. The following reagents were obtained from Beyotime Biotechnology (Shanghai, China): 4′,6‐diamidino‐2‐phenylindole (DAPI; C1005), β‐actin mouse monoclonal antibody (AF5001), and HRP‐labeled goat anti‐mouse IgG (H + L) (A0216). Additionally, the PRV glycoprotein B (gB)‐specific antibody and PRV‐positive serum were generated in the innovation team for prevention and control of swine and poultry important infectious diseases laboratory.

### 2.3. Cell Viability Assay

Cell toxicity of GLP was assessed using the Enhanced Cell Counting Kit‐8 in PK‐15 and Vero cells (CCK‐8, Beyotime, Shanghai, China). Briefly, cells were seeded in a 96‐well plate and treated with various concentrations of GLP (600, 800, 1000, and 1200 μg/mL) for 24 h at 37°C. Subsequently, 10 μL of CCK‐8 solution was added to each well, and the plates were incubated for an additional 2 h at 37°C. The absorbance was measured at a wavelength of 450 nm, and cell viability was calculated as a percentage relative to the untreated control group.

### 2.4. Infectivity Assay

PK‐15 cells were cultured in 6‐well plates under standard conditions at 37°C. When a confluence of 70%–80% was achieved, the existing medium was discarded, and the cells were rinsed three times with PBS prior to infection. The cells were pretreated with GLP (600, 800, 1000, and 1200 μg/mL) in DMEM at 37°C for 1 h. Expose PK‐15 cells to PRV XJ5 with 0.1 multiplicity of infection (MOI) at 37°C for 1 h. 1 h postinfection, the cells were washed three times with PBS and then incubated in DMEM with 2% FBS and the appropriate GLP concentrations at 37°C for 24 h. The antiviral properties of GLP were evaluated through western blot, 50% tissue culture infective dose (TCID_50_), and indirect immunofluorescence assay (IFA).

### 2.5. Time‐of‐Addition Assays

Time‐of‐addition experiments were performed to investigate the stage‐specific antiviral effects of GLP. PK‐15 cells were treated with GLP (600, 800, 1000, and 1200 μg/mL) at different stages relative to PRV XJ5 (MOI = 0.1) infection. The specific protocols were as follows: (i) Pretreatment: cells were incubated with GLP at 37°C for 1 h, subsequently washed thrice with PBS to remove the compound, and then infected with PRV for 1 h. Following infection, cells were washed again with PBS to remove unabsorbed virions. (ii) Cotreatment: cells were simultaneously exposed to PRV and the corresponding concentrations of GLP for 1 h at 37°C, followed by three washes with PBS to remove both the compound and unbound viruses. (iii) Posttreatment: cells were first infected with PRV for 1 h at 37°C and washed with PBS. GLP was then added, and the incubation continued at 37°C. In all groups, the treated cells were finally incubated in a fresh medium for an additional 24 h at 37°C.

### 2.6. Viral Attachment, Penetration, and Replication Assays

To assess the effect of GLP on viral attachment, PK‐15 cells were exposed to PRV XJ5 (MOI = 0.1) in the presence of GLP (600–1200 μg/mL) for 1 h at 4°C. Following the binding period, the cells were washed extensively with ice‐cold PBS and subsequently maintained in DMEM with 2% FBS for 24 h at 37°C.

In the viral penetration assay, PK‐15 cells were infected with PRV XJ5 (MOI = 0.1) for a 1 h period at 4°C and subsequently rinsed thrice with ice‐cold PBS to remove unbound viruses. Subsequently, the infected cells were exposed to GLP at concentrations of 600, 800, 1000, and 1200 μg/mL at 37°C for 1 h to halt internalization. Following a 1 h interval, we rinsed the infected cells three times with citric acid and then with PBS to eliminate PRV attached to the cell surface. The cells were then incubated at 37°C in DMEM with 2% FBS for 24 h.

During the viral replication experiment, PK‐15 cells were infected with PRV XJ5 (MOI = 0.1) for 1 h at 37°C without GLP and then rinsed three times with PBS. Afterward, the infected cells were incubated with GLP at doses of 600, 800, 1000, and 1200 μg/mL at 37°C. To measure the viral DNA, the cells were collected at 4 and 6 h postinfection using quantitative PCR.

### 2.7. Western Blot

The harvested cells were broken down using 4 × SDS‐PAGE loading buffer and heated in a metal bath at 96°C for 15 min. The samples separated using 12% SDS‐PAGE were moved from the gel to a nitrocellulose (NC) membrane. Blocking of the NC membrane was performed using PBST supplemented with 5% nonfat milk at room temperature for 2 h. The NC membrane was rinsed three times with PBST and left to incubate overnight at 4°C with either anti‐PRV gB monoclonal antibodies at a 1:200 dilution or actin monoclonal antibodies at a 1:5000 dilution. After overnight incubation, the NC membrane was thoroughly washed three times with PBST to remove the unbound primary antibodies. It was then incubated with HRP‐conjugated secondary antibodies (1:5000 dilution) for 2 h at room temperature. For the final detection, the specific protein‐antibody complexes were visualized using an enhanced chemiluminescence (ECL) detection kit from Share Biotechnology (Shanghai, China).

### 2.8. Viral Titer Assay

The TCID_50_ assay was employed to quantify viral titers. PK‐15 cells, prepared in 96‐well plates, were inoculated with serially diluted virus (10^−1^ to 10^−7^ in DMEM) for 1.5 h at 37°C to allow viral adsorption. The inoculum was then aspirated, and the cells were overlaid with fresh DMEM containing 2% FBS for a 72 h incubation period at 37°C. The resulting cytopathic effect (CPE) was scored, and the TCID_50_ value was determined according to the Reed–Muench method.

### 2.9. IFA

Following fixation with 4% PFA (37°C, 30 min) and permeabilization with 0.1% Triton X‐100 (37°C, 10 min), cells were blocked overnight at 4°C with 5% BSA. The next day, cells were incubated with PRV‐positive serum (1:200) and the FITC‐labeled secondary antibody (rabbit anti‐pig IgG) for 1 h and 30 min at 37°C, respectively, with three PBS washes performed prior to each step. The nuclei were then stained with DAPI for 7 min, and images were acquired using a fluorescence microscope.

### 2.10. Q‐PCR

The phenol chloride method was used to extract total DNA from cells or cell supernatants [18]. Viral DNA was measured using q‐PCR. In brief, gene level was assessed with the following specific primers: gB‐F: ACAAGTTCAAGGCCCACATCTAC (forward) and gB‐R: GTCCGTGAAGCGGTTCGTGAT (reverse). The procedure involved heating at 95°C for 60 s, then 45 cycles of 95°C for 10 s, and finally 62°C for 20 s.

### 2.11. Statistical Analysis

The results were derived from three separate experiments and are presented as the means ± SD. Each experimental group was compared to the group with 0 concentration. Using one‐way ANOVAs, all data were analyzed with GraphPad Prism software. Significance levels were as follows: ns, not significant;  ^∗^ *p* < 0.05;  ^∗∗^ *p* < 0.01; and  ^∗∗∗^ *p* < 0.001.

## 3. Result

### 3.1. Toxicity of GLP Toward PK‐15 and Vero Cells

The potential cytotoxicity of GLP (600, 800, 1000, and 1200 μg/mL) was first examined in PK‐15 and Vero cells using the enhanced CCK‐8 method to determine the appropriate treatment concentrations. Compared with the negative control group, results revealed no cytotoxic effects of GLP from 600 to 1200 μg/mL (Figure 1A, B). Therefore, GLP concentrations of 600–1200 μg/mL were selected for the following experiments.

**Figure 1:**
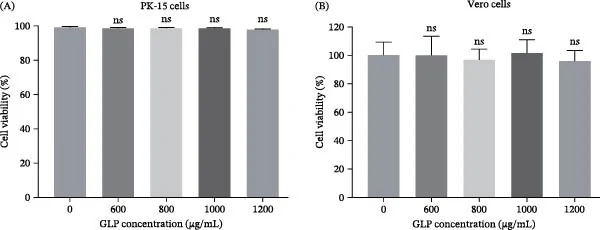
GLP’s cytotoxic effects on PK‐15 and Vero cells were identified using the enhanced CCK‐8. (A and B) PK‐15 and Vero cells were incubated with varying levels of GLP for 24 h, followed by the addition of 10 μL CCK‐8 solution for an additional 2 h. Absorbance was then read at OD_450_ nm (ns, not significant).

### 3.2. GLP Inhibits PRV Infection Within PK‐15 and Vero Cells

The antiviral activity of GLP against PRV infection was subsequently assessed by exposing infected PK‐15 cells to increasing concentrations of GLP for 1 h. The cells were subsequently infected with PRV XJ5 (MOI = 0.1) in the continued presence of GLP for another hour. Following infection, the cells were washed three times with PBS and then maintained in DMEM containing 2% FBS and the corresponding concentrations of GLP (600, 800, 1000, and 1200 μg/mL) at 37°C for 24 h. The western blot analysis showed that PRV gB protein expression decreased at GLP concentrations ranging from 600 to 1200 μg/mL (Figure 2A, B). Additionally, the virus titer in the cell supernatant was assessed, revealing that GLP decreased virion production in a dose‐dependent manner (Figure 2C). According to IFA results, an increase in GLP concentration led to the reduction in the number of infected cells (Figure 2D). In addition, we found GLP inhibited PRV gB protein expression in Vero cells (Figure 2E, F).

**Figure 2:**
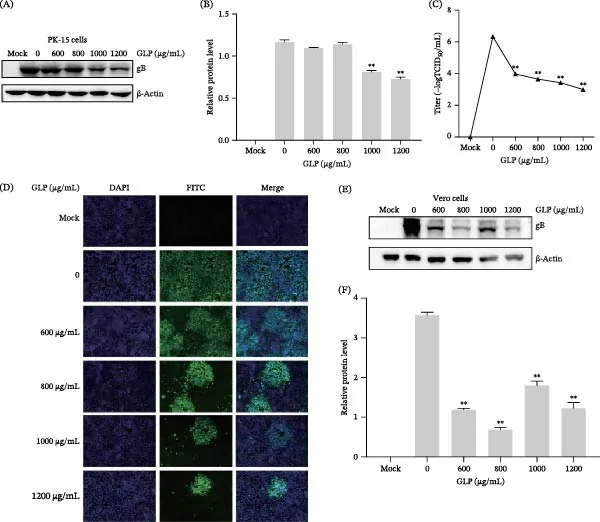
GLP inhibited PRV infection. (A) Protein levels of PRV gB protein and β‐actin analyzed by western blot in PK‐15 cells. (B) Ratio of PRV gB to β‐actin by densitometric analysis. (C) The viral titer in collected supernatants was quantified by TCID_50_. (D) The internalized virus was detected by immunofluorescence assay. (E) Protein levels of PRV gB protein and β‐actin analyzed by western blot in Vero cells. (F) Ratio of PRV gB to β‐actin by densitometric analysis. All results were obtained from three separate assays and were presented in means ± SD ( ^∗∗^ *p* < 0.01).

### 3.3. GLP Affects PRV Attachment and Penetration Into PK‐15 and Vero Cells

To explore the antiviral effect of GLP at the initial stage of PRV infection, we treated cells with different GLP concentrations (600, 800, 1000, and 1200 μg/mL) in the presence of PRV XJ5 (MOI = 0.1) at 4°C for 1 h. The cells were rinsed three times with precooled PBS, followed by 1 h incubation with GLP at 37°C. Subsequently, they were washed three times with citric acid solution and PBS to eliminate uninternalized viral particles. Finally, we cultivated the cells with DMEM containing 2% FBS at 37°C. At 24 hpi, viral titers in the supernatants were measured using TCID_50_, while PRV gB protein expression was analyzed by western blot. Additionally, the number of infected cells were evaluated by IFA. The result showed that the protein level of PRV gB reduced, especially with 1000 and 1200 μg/mL GLP (Figure 3A, B). The collected supernatants revealed that PRV titer decreased dose dependently (Figure 3C). According to IFA assay, GLP decreased the number of infected cells (Figure 3D). Q‐PCR revealed that GLP reduced PRV DNA levels (Figure 3E). In addition, we found that GLP inhibited PRV gB protein levels in Vero cells (Figure 3F, G). The findings indicate that GLP inhibits the initial phases of PRV infection.

**Figure 3:**
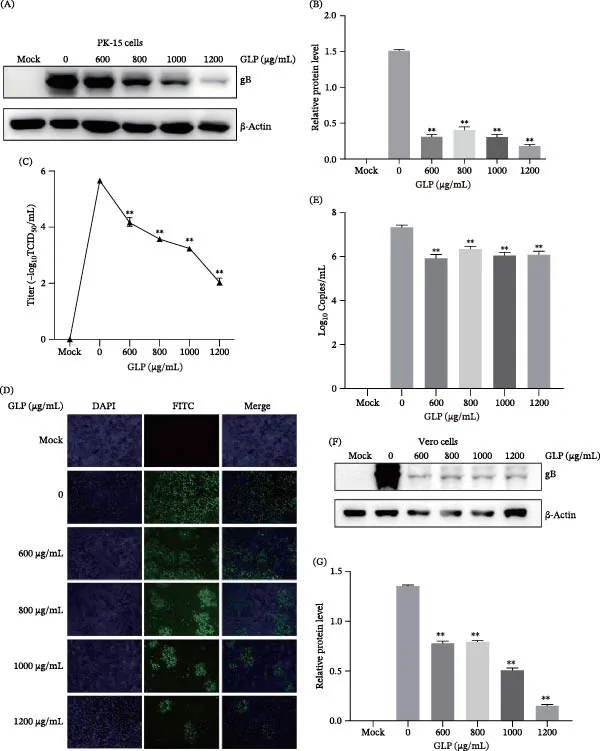
GLP Affects PRV attachment and penetration into PK‐15 and Vero cells. (A and B) Western blot for detection of gB expression after treatment with GLP in PK‐15 cells. (C) TCID_50_ for detection of viral titer after treatment with GLP. (D) Immunofluorescence assay for detection of internalized virus after treatment with GLP. (E) The copy numbers of PRV DNA were quantified by q‐PCR. (F and G) Western blot for detection of gB expression after treatment with GLP in Vero cells. All results were obtained from three separate assays and were presented in means ± SD ( ^∗∗^ *p* < 0.01).

### 3.4. GLP Inhibits PRV Binding to PK‐15 and Vero Cells

To determine if GLP interferes with PRV attachment to the cell surface, cells were incubated with PRV XJ5 (MOI = 0.1) and GLP at 4°C for 1 h to allow binding while blocking internalization. Following incubation, the cells were thoroughly rinsed with ice‐cold PBS and then cultured at 37°C. The efficiency of viral binding was evaluated at 24 hpi by detecting PRV gB protein levels via western blot. The results revealed that the gB protein level of cells treated by GLP decreased, obviously indicating that GLP almost completely blocked the binding of PRV particles to the surface of host cell membranes (Figure 4A, B). In the same way, according to the TCID_50_ assay and IFA assay, GLP decreased the PRV titer and the numbers of infected cells (Figure 4C, D). Q‐PCR reveled that GLP reduced PRV DNA levels (Figure 4E). In addition, we found that GLP inhibited PRV gB protein levels in Vero cells (Figure 4F, G).

**Figure 4:**
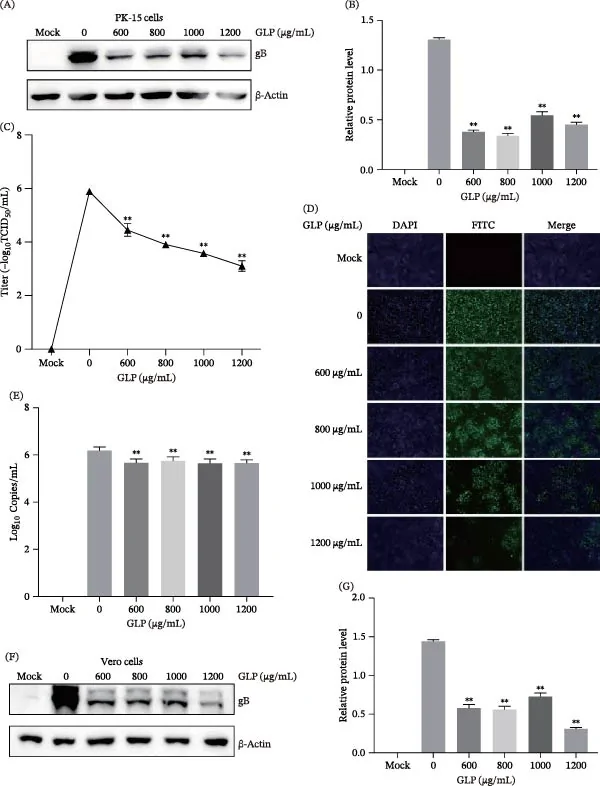
GLP inhibits PRV binding to PK‐15 and Vero cells. (A and B) At 24 hpi, the expression levels of gB protein and actin were analyzed by western blot. (C) TCID_50_ was used to detect virus titer. (D) The internalized virus was detected by immunofluorescence assay. (E) The copy numbers of PRV DNA were quantified by q‐PCR. (F and G) Western blot for detection of gB expression after treatment with GLP in Vero cells. All results were obtained from three separate assays and were presented in means ± SD ( ^∗∗^ *p* < 0.01).

### 3.5. Effect of GLP on PRV Internalization

To investigate whether GLP inhibits PRV penetration into PK‐15 and Vero cells following viral attachment, we infected cells with DMEM containing PRV XJ5 (MOI = 0.1) only for 1 h at 4°C followed by washing thrice with cold PBS. Then, we treated them with the corresponding concentration of GLP (600, 800, 1000, and 1200 μg/mL) for 1 h at 37°C. To inactivate and remove virions that had not been internalized, the cells were treated with citric acid solution and washed with PBS before being maintained in 2% FBS‐DMEM. At 24 hpi, the western blot and IFA assays indicated that GLP had barely no effect on the penetration of PRV at 600, 800, and 1000 μg/mL, only decreased the penetration of PRV at 1200 μg/mL (Figure 5A, B, D). The TCID_50_ assay result showed that GLP decreased the viral titer after treatment by GLP (Figure 5C). Q‐PCR reveled that GLP had no effect on PRV DNA levels (Figure 5E). In addition, we found that GLP only inhibited PRV gB protein levels at 1200 μg/mL in Vero cells (Figure 5F, G).

**Figure 5:**
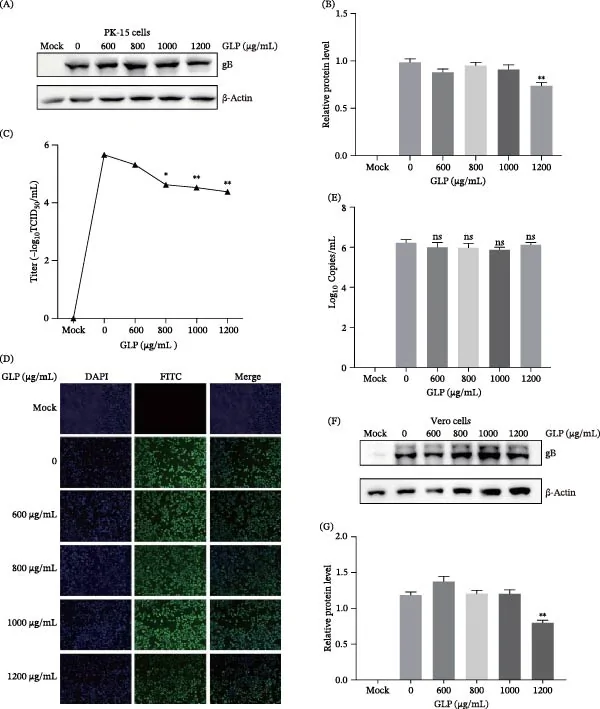
Effect of GLP on PRV Internalization. (A and B) At 24 h postinfection, western blot was used to analyze the expression levels of gB protein and actin in PK‐15 cells. (C) The virus titer was determined using the TCID_50_ method. (D) The internalized virus was detected by immunofluorescence assay. (E) The copy numbers of PRV DNA were quantified by q‐PCR. (F and G) Western blot for detection of gB expression after treatment with GLP in Vero cells. All data are representative of three independent experiments and are expressed as the mean ± standard deviation (SD). Statistical significance is denoted as follows:  ^∗^ *p* < 0.05;  ^∗∗^ *p* < 0.01.

### 3.6. Effect of GLP on PRV Replication

To assess the effect of GLP on viral replication, PK‐15 and Vero cells were first infected with PRV XJ5 (MOI = 0.1) for 1 h at 37°C. The inoculum was then removed by washing the cells three times with PBS, and the infected cells were thereafter treated with specific concentrations of GLP in DMEM at 37°C. To evaluate viral DNA levels, the cells were washed and collected at 4 and 6 h postinfection for q‐PCR and western blot measurement. We found that the viral DNA copy level decreased at 4 and 6 hpi (Figure 6A, B). Western blot revealed that GLP inhibited PRV gB protein levels at 4 and 6 hpi in PK‐15 (Figure 6C–F) and Vero cells (Figure 6G–J). These results indicating that GLP had the effect on PRV replication.

**Figure 6:**
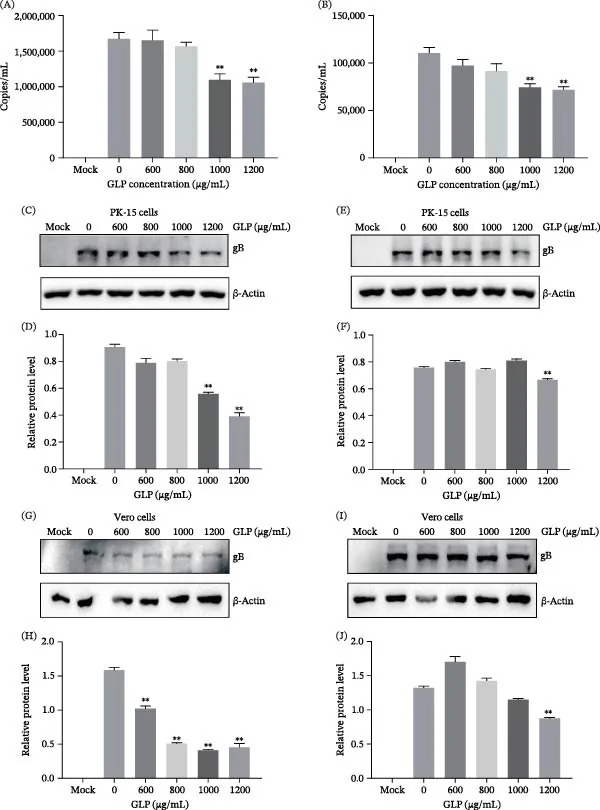
GLP had an effect on PRV replication. PK‐15 cells were infected with PRV XJ5 (MOI = 0.1) at 37°C for 1 h, then incubated with GLP at 37°C. (A) At 4 hpi, the copy numbers of PRV DNA were quantified by q‐PCR. (B) At 6 hpi, the copy numbers of PRV DNA were quantified by q‐PCR. (C and D) At 4 hpi, PRV gB were checked by western blot at Pk‐15 cells. (E and F) At 6 hpi, PRV gB were checked by western blot at Pk‐15 cells. (G and I) At 4 hpi, PRV gB were checked by western blot at Vero cells. (H and J) At 6 hpi, PRV gB were checked by western blot at Vero cells. All results were obtained from three separate assays and were presented in means ± SD ( ^∗^ *p* < 0.05;  ^∗∗^ *p* < 0.01).

## 4. Discussion

In 2011, PRV variant appeared in numerous Bartha K61‐vaccinated pig herds in China, garnering increasing attention because of its significant threat to both domestic and wild animals, as well as humans [19]. At present, the Bartha K61 live attenuated vaccine is employed globally to protect pigs from PRV infection [20]. Nonetheless, increasing evidence suggests that these vaccines do not offer full protection against new PRV variants that have appeared since late 2011 [21]. Chinese herbal medicine, with the history of thousands of years, has been widely used to treat various human diseases and has also shown potential in improving certain animal diseases [22]. In previous studies, many herbs have been studied for their antiviral effects against PRV, such as (‐)‐epigallocatechin‐3‐gallate, which inhibits PRV infection by inhibiting virus replication and adsorption [18]. Resveratrol, flos Lonicerae japonica extract, and luteolin inhibit PRV by targeting the NF‐κB signaling pathway [23–25]. Quercetin inhibits PRV infection by decreasing the binding activity of gD [26]. Platycodon grandifloras polysaccharides inhibit PRV infection by targeting the Akt/mTOR signaling pathway [27]. In a word, traditional Chinese herbal medicines hold significant potential in inhibiting PRV infection and require further research to become a viable option for combating PRV in animals or humans. This study provides the first demonstration that GLP effectively inhibits PRV infection in vitro.

*Ganoderma lucidum* is considered as a potential candidate for the development of various antiviral agents. Liposomes encapsulating GLP (Lip‐PS) were found to induce a more robust PCV‐II‐specific immune response compared to other single‐component formulations [28]. A virtual screening approach followed by an in vitro assay predicted the functional triterpenoids from *Ganoderma lucidum* as potential inhibitors of DENV NS2B‐NS3 protease against dengue virus [29]. *Ganoderma lucidum* can be used to help in the treatment of COVID‐19 infections by supplying patients with COVID‐19, and protective inflammatory responses were developed [30]. Therefore, GLP was selected to explore its antiviral activity against PRV.

Membrane fusion is the method by which PRV infects cells, with the viral glycoprotein gD playing a role in the attachment of PRV to cells after the loose interaction between gC and the cell surface’s heparan sulfate [31]. The integral membrane gH and anchorless gL form the heterodimeric complex, and along with the homotrimeric gB, they are involved in the core PRV envelope fusion with the cellular cytoplasmic membrane [32]. Ultimately, the PRV genomic DNA inside the cell is released from the structurally intact capsid into the host nucleus, where it is replicated [33]. Therefore, in this study, we made schedules to explore whether GLP has inhibition on these stages. Our study confirmed that GLP caused a decrease in PRV infectivity. It can strongly inhibit PRV binding to the surface of PK‐15 cells, but its intracellular entry remained largely unaffected.

## 5. Conclusion

In conclusion, this work identifies GLP as a previously unexplored inhibitor of PRV. The anti‐PRV activity of GLP is exerted through the disruption of two distinct early stages of infection: viral adsorption and genome replication. The research suggests a possible treatment option for combating PRV infection, though the in vivo antiviral effects of GLP need more investigation.

## Funding

The project was funded by the Priority Academic Program Development of Jiangsu Higher Education Institutions (PAPD), and it was supported by the 111 Project (Grant D18007) and the Top‐level Talents Support Program of Yangzhou University.

## Ethics Statement

This study involved only in vitro experiments using established animal cell lines maintained in our laboratory. No live animals or human participants were involved; therefore, ethical approval was not required.

## Conflicts of Interest

The authors declare no conflicts of interest.

## Data Availability

The data that support the findings of this study are available from the corresponding author upon reasonable request.
